# Antimicrobial-resistant *Staphylococcus* spp. and *Mammaliicoccus sciuri* isolated from rehabilitating wild mammals in northeastern Brazil: implications for microbiological surveillance and biosafety in wildlife care facilities

**DOI:** 10.1007/s11259-026-11465-0

**Published:** 2026-08-26

**Authors:** Denny Parente de Sá Barreto Maia Leite, Taoana Perrelli Sarmento, Valdir Vieira da Silva, Lucilene Martins Trindade Gonçalves, Pollyanne Raysa Fernandes de Oliveira, Rafaela Silva Santos, Maria Eduarda Uchôa Cavalcanti Moreira da Silva, Gustavo de Oliveira Alves Pinto, José Givanildo da Silva, Karolina Rosa Fernandes Beraldo, Maria Aparecida Juliano, Rinaldo Aparecido Mota

**Affiliations:** 1 Department of Veterinary Medicine, Federal Rural University of Pernambuco, Recife, Pernambuco Brazil; 2 Department of Preventive Veterinary Medicine and Animal Production, School of Veterinary Medicine and Animal Science, Federal University of Bahia, Salvador, Bahia Brazil; 3 Department of Biophysics, Paulista School of Medicine), Federal University of São Paulo, São Paulo, Brazil

**Keywords:** Antimicrobial resistance, Wildlife rehabilitation, Resistance genes, Biofilm formation, Biosafety

## Abstract

Antimicrobial resistance (AMR) among *Staphylococcus* and *Mammaliicoccus* species is a growing concern in wildlife care settings, as rehabilitation centers may represent contact points among wild animals, captive environments, and human handlers. This study characterized antimicrobial resistance profiles, multiple antibiotic resistance (MAR) indices, and biofilm formation in *Staphylococcus* spp. and *Mammaliicoccus sciuri* isolated from wild mammals housed at a wildlife screening and rehabilitation center in Pernambuco, northeastern Brazil. Oropharyngeal and rectal swabs were collected from 84 individuals representing 15 species. Isolates were identified using MALDI-TOF MS, and susceptibility to oxacillin, cefoxitin, clindamycin, erythromycin, gentamicin, norfloxacin, and tetracycline was assessed by disk diffusion. Multidrug resistance (MDR) and MAR indices were determined, and the resistance genes *mecA*, *mecC*, *norA*, *msrA*, *tet(38)*, and *tetM* were investigated by PCR. Bacterial growth on mannitol salt agar was obtained from samples collected from 63/84 (75.0%) animals, yielding 63 confirmed isolates. *Mammaliicoccus sciuri* was the most frequently identified species, accounting for 29/63 (46.0%) isolates, followed by *Staphylococcus aureus*, *Staphylococcus felis*, and *Staphylococcus simulans*, with 5/63 (7.9%) isolates each. Overall, 50/63 (79.4%) isolates were resistant to at least one antimicrobial category, and 13/63 (20.6%) were classified as MDR. Resistance was most frequently observed to oxacillin (68.3%), followed by cefoxitin (28.6%), erythromycin (27.0%), tetracycline (25.4%), clindamycin (22.2%), norfloxacin (11.1%), and gentamicin (11.1%). MAR indices ranged from 0.00 to 1.00, with a mean value of 0.28. Resistance genes were detected in a subset of isolates, including *mecA* (4/63; 6.3%), *norA* (2/63; 3.2%), and *msrA* (1/63; 1.6%), whereas *mecC*, *tet(38)*, and *tetM* were not detected. Biofilm formation was observed in 33/63 (52.4%) isolates, predominantly among weak and moderate producers. These findings provide baseline evidence supporting systematic microbiological surveillance, antimicrobial stewardship, and biosafety measures in wildlife rehabilitation facilities within a One Health framework.

**Graphical abstract:**

**Figure:**
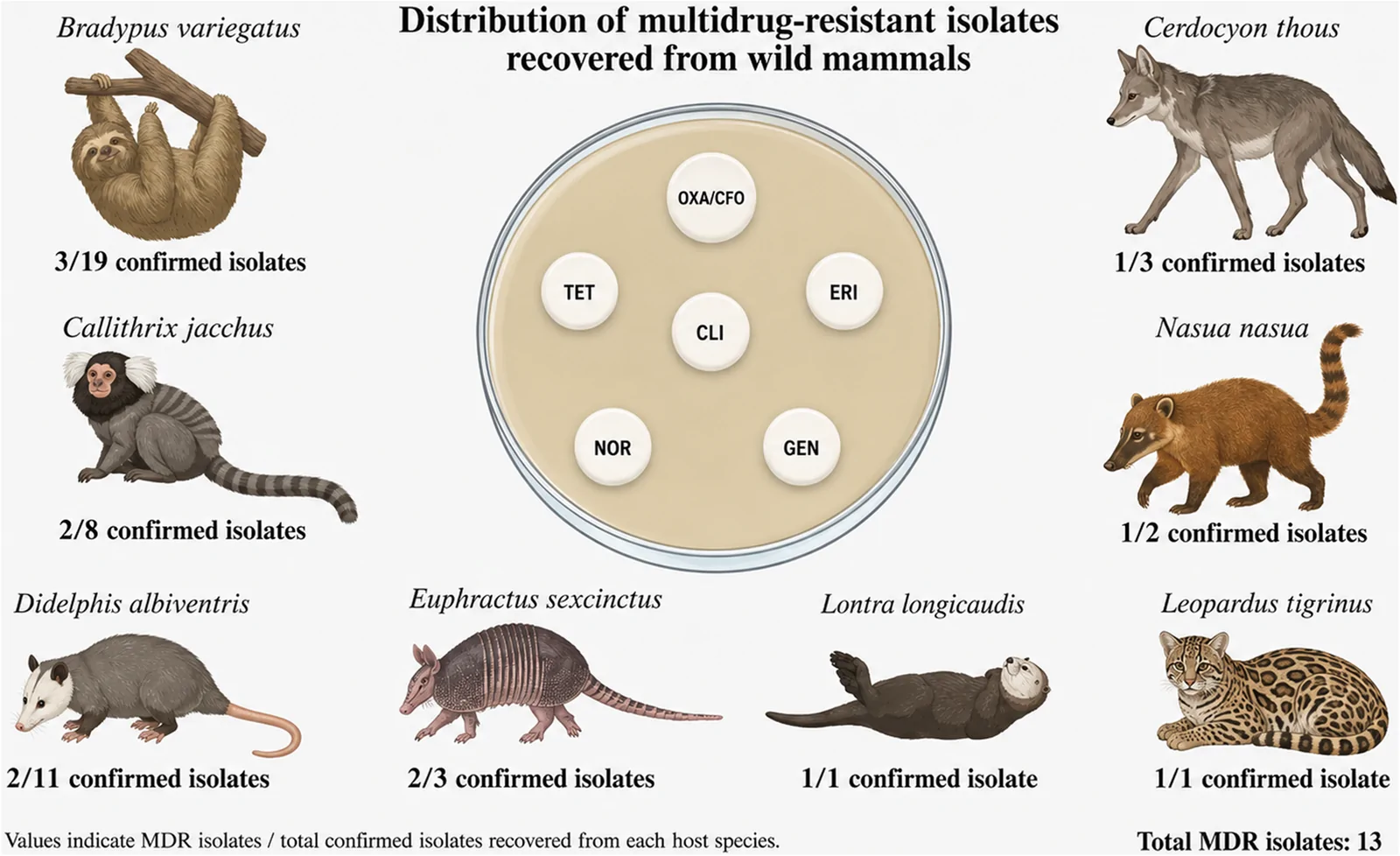

**Supplementary Information:**

The online version contains supplementary material available at https://doi.org/10.1007/s11259-026-11465-0.

##  Introduction

Antimicrobial resistance (AMR) is recognized as one of the greatest threats to global health and directly affects the human–animal–environment interface within the One Health framework. Among the microorganisms of concern, members of the *Staphylococcus* genus are particularly relevant, especially methicillin-resistant *Staphylococcus aureus* (MRSA), an important target of AMR surveillance (WHO 2014; WAHIS 2016).

Staphylococci are Gram-positive, facultatively anaerobic cocci that include coagulase-positive species, such as *Staphylococcus aureus* and *Staphylococcus pseudintermedius*, and coagulase-negative species, such as *Staphylococcus epidermidis* and *Staphylococcus haemolyticus* (Sousa et al. 2014; Bagheri et al. 2019). Members of the *Mammaliicoccus sciuri* group, formerly classified within the genus *Staphylococcus*, are also recognized as colonizers and potential opportunistic pathogens. Although these microorganisms may be part of the normal microbiota of humans and animals, they can cause conditions ranging from mild skin infections to mastitis, endocarditis, and septicemia (Xenoulis et al. 2010; Bagheri et al. 2019).

The misuse and overuse of antimicrobials in human medicine, veterinary practice, and animal production, together with pharmaceutical waste and environmental changes, contribute to the selection and dissemination of resistant bacteria across human, animal, and environmental compartments (Holmes et al. 2016; Chatterjee et al. 2018; Matias et al. 2018; Garcia et al. 2020).

Wild animals undergoing rehabilitation deserve particular attention because they may originate from anthropized environments, illegal captivity, or other circumstances involving previous contact with humans (Tardón et al. 2021). Antimicrobial-resistant bacteria have been isolated from wounds and open fractures in wild birds admitted to rehabilitation centers (Sánchez-Ortiz et al. 2024). Resistant bacteria have also been detected in animals and environmental surfaces within a wildlife rehabilitation center, although transmission between these sources was not demonstrated (Baros Jorquera et al. 2021).

In captive mammals, genomic evidence has indicated the circulation of antimicrobial-resistant *Escherichia coli* among species housed in separate enclosures (Sealey et al. 2023). Handling, confinement, and clinical interventions are recognized stressors in wild animals undergoing rehabilitation (Huber et al. 2019; Narayan and Vanderneut 2019; Charalambous et al. 2021). However, stress, close animal proximity, shared care environments, and frequent handling influence the persistence or transmission of antimicrobial-resistant bacteria in rehabilitating wild mammals remains unknown. This possibility should therefore be regarded as a hypothesis requiring further epidemiological and genomic investigation.

Antimicrobial-resistant bacteria have been reported in wildlife from diverse ecological contexts, including Brazil and other Latin American countries (Pérez-Maldonado et al. 2025). However, information on animals undergoing rehabilitation remains limited. Because rehabilitation centers temporarily bring together animals from different origins, these facilities may provide valuable opportunities for microbiological surveillance and for improving infection-control and biosafety practices (Sacristán et al. 2020).

This study aimed to characterize antimicrobial resistance and biofilm formation in *Staphylococcus* spp. and *M. sciuri* isolated from wild mammals undergoing rehabilitation at the Pernambuco Wild Animal Triage and Rehabilitation Center (CETRAS–Tangara). Phenotypic and genotypic resistance mechanisms were investigated to support microbiological surveillance and biosafety strategies in wildlife rehabilitation facilities.

## Materials and methods

### Study area and biological samples

The study was conducted at the Pernambuco Wild Animal Triage and Rehabilitation Center (CETRAS–Tangara), located in Recife, northeastern Brazil. The facility receives wild animals admitted under various circumstances, including seizure from illegal wildlife trade, rescue of free-ranging animals in vulnerable conditions, voluntary surrender, and other situations requiring temporary care and rehabilitation (IBAMA 2020).

Samples from the wild mammals included in this study were collected in 2024. Microbiological and molecular analyses were subsequently conducted between 2024 and 2025 at the Laboratory of Infectious Disease Diagnosis of the Universidade Federal Rural de Pernambuco. During routine animal handling procedures at the rehabilitation center, oropharyngeal and rectal samples were collected using sterile swabs. The inclusion of host species was determined by the availability of animals on the sampling days and authorization from the attending veterinary team, reflecting the operational routine of the facility.

Sampling followed an opportunistic, non-probabilistic design rather than a longitudinal or prospectively stratified protocol. Animals were sampled whenever access was authorized and sample collection was considered operationally feasible under veterinary supervision. Eligibility was assessed during each sampling visit according to animal availability, routine care priorities, and welfare considerations. Consequently, the total number of potentially eligible animals that were not sampled was not prospectively recorded and could not be reliably reconstructed.

Only animals considered clinically stable enough to undergo handling, as determined by the attending veterinarians, were included. For this study, “clinically stable” referred to animals without immediate life-threatening conditions, severe debilitation, marked prostration, or any clinical condition in which restraint and swab collection could interfere with urgent medical care or increase the risk of clinical deterioration.

This criterion did not necessarily indicate that the animals were healthy, but rather that they were considered suitable for brief handling without compromising their welfare or ongoing treatment. Animals receiving antimicrobial therapy at the time of sampling were excluded. When previous antimicrobial treatment was documented in the clinical records, sampling was performed only after at least 30 days had elapsed since treatment completion.

The interval between admission to CETRAS–Tangara and sample collection was not standardized because animals were sampled at different stages of rehabilitation according to the facility’s routine and veterinary authorization. Individual records concerning origin, prior captivity, reason for admission, and length of stay were not consistently complete for all sampled animals. Therefore, these variables were not included in comparative analyses, and the lack of standardized individual-level contextual information was considered when interpreting the findings.

Samples were collected from 84 wild mammals, yielding 168 biological samples, comprising one oropharyngeal and one rectal swab from each animal. The taxonomic classification of the sampled animals according to order, family, species, and common English name is presented in Table 1.

**Table 1:** Number of mammals sampled according to order, family, common name, and species

Order	Family	Popular name	Species	No. of animals
Carnívora	*Canidae*	Crab-eating fox	*Cerdocyon thous*	3
	*Felidae*	Northern tiger cat	*Leopardus tigrinus*	1
		Jaguarundi	*Puma yagouaroundi*	4
	*Mustelidae*	Neotropical otter	*Lontra longicaudis*	1
	*Procyonidae*	Crab-eating raccoon	*Procyon lotor*	1
		South American coati	*Nasua nasua*	3
Cingulata	*Dasypodidae*	Six-banded armadillo	*Euphractus sexcinctus*	3
Didelphimorphia	*Didelphidae*	White-eared opossum	*Didelphis albiventris*	27
Pilosa	*Bradypodidae*	Brown-throated sloth	*Bradypus variegatus*	19
	*Myrmecophagidae*	Southern tamandua	*Tamandua tetradactyla*	6
Primates	*Cebidae*	Bearded capuchin	*Sapajus libidinosus*	2
	*Callitrichidae*	Common marmoset	Callithrix jacchus	8
Rodentia	*Caviidae*	Rock cavy	*Kerodon rupestris*	1
		Brazilian guinea pig	*Cavia aperea*	1
	*Erethizontidae*	Baturité porcupine	*Coendou speratus*	4
Total				**84**

Each sample was labeled and placed in a tube containing Mueller–Hinton (MH) broth supplemented with 7.5% sodium chloride. The increased salinity was intended to favor the recovery of halotolerant microorganisms, including members of the genera *Staphylococcus* and *Mammaliicoccus* (Quinn et al. 2016). The tubes were then transported under refrigerated conditions (4–10 °C) in insulated containers with reusable ice packs to the Laboratory of Infectious Disease Diagnosis at the Universidade Federal Rural de Pernambuco, where the microbiological and molecular analyses were performed.

### Microbiological analysis

In the laboratory, the oropharyngeal and rectal swabs were processed independently. Samples in Mueller–Hinton (MH) broth were initially incubated at 37 °C for 24 h. Bacterial isolation was subsequently performed by plating onto mannitol salt agar (MSA), followed by incubation at 37 °C for 24–48 h (Leite et al. 2023).

After incubation, the plates were macroscopically examined for colonies compatible with members of the family Staphylococcaceae. When homogeneous colony was observed, one representative colony was selected. When two or more macroscopically distinct morphotypes compatible with the target bacterial group were present on the same plate, one representative colony of each morphotype was selected and processed separately.

Selected colonies were subjected to Gram staining, morphological assessment, and catalase testing (Quinn et al. 2016). After purification, presumptive isolates were identified at the species level using matrix-assisted laser desorption/ionization time-of-flight mass spectrometry (MALDI-TOF MS), according to the protocol described by Clark et al. (2013). Distinct morphotypes recovered from the same sample were retained as separate isolates when they were identified as different bacterial species.

Isolates identified as *Staphylococcus* spp. or *Mammaliicoccus sciuri* were subcultured onto fresh MSA plates. A portion of the bacterial material was used for DNA extraction, while the remaining material was inoculated into brain heart infusion (BHI) broth, incubated at 37 °C for 24 h, and stored at − 80 °C in 20% glycerol for long-term preservation (Leite et al. 2025).

The final analytical dataset consisted of confirmed *Staphylococcus* spp. and *M. sciuri* isolates recovered from independently processed oropharyngeal and rectal samples.

###  Antimicrobial susceptibility testing

Phenotypic antimicrobial resistance was assessed using the disk diffusion method. Bacterial suspensions were standardized to a 0.5 McFarland turbidity standard and inoculated onto Mueller–Hinton agar plates. Testing was performed according to the recommendations of the Clinical and Laboratory Standards Institute (CLSI) and the European Committee on Antimicrobial Susceptibility Testing (EUCAST). *Staphylococcus aureus* ATCC 25,923 and *Escherichia coli* ATCC 25,922 were used as quality-control strains (CLSI 2024; EUCAST 2025).

The antimicrobial panel included oxacillin (OXA, 1 µg), cefoxitin (CFO, 30 µg), clindamycin (CLI, 2 µg), erythromycin (ERI, 15 µg), gentamicin (GEN, 10 µg), norfloxacin (NOR, 10 µg), and tetracycline (TET, 30 µg). Commercial antimicrobial disks manufactured by Laborclin (Brazil) were used. The panel was selected based on the antimicrobials routinely used at the rehabilitation center, their relevance to veterinary practice, and their correspondence with the resistance genes investigated. Inhibition zone diameters were interpreted according to CLSI VET01S, 7th edition (CLSI 2024), and EUCAST recommendations (EUCAST 2025).

Because species-specific breakpoints are not available for *Mammaliicoccus sciuri*, interpretative criteria established for coagulase-negative staphylococci were applied (Leite et al. 2025).

Isolates were classified as multidrug-resistant (MDR) when they exhibited non-susceptibility to at least one antimicrobial agent in three or more antimicrobial categories (Magiorakos et al. 2012; Sweeney et al. 2019). For MDR classification, oxacillin and cefoxitin were considered representatives of the same β-lactam category and were therefore counted only once.

The multiple antibiotic resistance (MAR) index was calculated according to Krumperman (1983) using the formula a/b, where “a” represents the number of antimicrobial categories to which an isolate was resistant and “b” represents the total number of antimicrobial categories tested. Oxacillin and cefoxitin were considered within a single β-lactam category for this calculation.

###  DNA extraction and genotypic antimicrobial resistance profiling

Genomic DNA was extracted from bacterial colonies using the thermal lysis method described by Fan et al. (1995). Following extraction, DNA concentration and purity were assessed by spectrophotometry at 260 nm using a Thermo Fisher Scientific spectrophotometer (Massachusetts, USA) (Brakstad et al. 1992). PCR assays were performed for all confirmed isolates included in the study (*n* = 63), regardless of their phenotypic antimicrobial susceptibility profiles. Each target gene was investigated using a separate singleplex PCR assay; therefore, no multiplex reactions were performed. For each target gene, the complete PCR protocol, including the reaction mixture, primer concentrations, reaction volume, thermal cycling conditions, annealing temperature, and amplicon detection procedures, followed exactly the corresponding protocol cited in Table 2, without methodological modifications.

**Table 2:** Investigated resistance genes, primer sequences, and corresponding references

β-lactam resistance	Genes	Primers sequences (5’-3’)	References
	mecA	*R*: CTAATCTCATATGTGTTCCTGTATTGGC F: TGGTATGTGGAAGTTAGATTGGGAT	Nakagawa et al. (2005)
mecC	*R*: TGGCTGAACCCATTTTTGAT F: CATTAAAATCAGAGCGAGGC	Paterson et al. (2012)	
Efflux Pumps	*norA*	R: AGATTGCAATTCATGCTAAATATT F: TGCAATTTCATATGATCAATCCC	Truong-Bolduc et al. (2003)
	*msrA*	R: GCAAATGGTGTAGGTAAGACAACT F: ATCATGTGATGTAAACAAAAT	Wondrack et al. (1996)
	*tet(38)*	R: CGTAGAAATAAATCCACCTG F: TTCAGTTTGGTTATAGACAA	Truong-Bolduc et al. (2005)
Tetracycline Resistance	*tetM*	R: CGGTAAAGTTCGTCACACAC F: GTGGACAAAGGTACAACGAG	Ng et al. (2001)

The genes investigated included *mecA* and *mecC*, associated with β-lactam resistance; *norA*, associated with fluoroquinolone efflux; *msrA*, associated with resistance to macrolides and group B streptogramins; and *tet(38)* and *tetM*, associated with tetracycline resistance.

###  Phenotypic evaluation of biofilm formation

Biofilm production was evaluated using modified protocols originally described by Pratt and Kolter (1998) and Stepanovic et al. (2000). Isolates were initially cultured on tryptic soy agar (TSA) at 37 °C for 24 h and subsequently inoculated into tryptic soy broth (TSB) supplemented with 0.1% glucose, followed by incubation under the same conditions.

Bacterial suspensions were standardized to a 0.5 McFarland turbidity standard. Aliquots of 100 µL of each bacterial suspension and 100 µL of glucose-supplemented TSB were dispensed in triplicate into 96-well microtiter plates and incubated for 24 h. After incubation, the wells were washed, air-dried, and stained with 0.25% crystal violet. The retained dye was subsequently solubilized using an alcohol–acetone solution (80:20), and absorbance was measured at 620 nm.

*Escherichia coli* ATCC 35,218, previously described as a weak biofilm-producing strain, was used as a procedural positive control for bacterial adherence and crystal-violet staining rather than as a reference for strong biofilm production. Sterile culture medium was used as the negative control.

The optical density cut-off value (ODc) was defined as the mean OD of the negative-control wells plus three standard deviations. Biofilm production was classified according to the OD of each isolate relative to the ODc as follows: OD ≤ ODc, non-producer; ODc < OD ≤ 2 × ODc, weak producer; 2 × ODc < OD ≤ 4 × ODc, moderate producer; and OD > 4 × ODc, strong producer.

###  Statistical analysis

Given the opportunistic, non-probabilistic sampling design and the limited and uneven numbers of animals across host species, the data was analyzed using descriptive statistics. At the host-species level, results were primarily summarized using absolute frequencies, including the numbers of animals sampled, animals yielding confirmed isolates, isolates recovered, bacterial species identified, and anatomical sampling sites.

Percentages representing antimicrobial resistance or isolate occurrence within individual host species were not interpreted as prevalence estimates because of the small and uneven sample sizes. Percentages were used for overall isolate-level summaries, including antimicrobial resistance profiles, resistance genes, MAR indices, and biofilm production categories.

Antimicrobial susceptibility results were summarized as absolute and relative frequencies of resistant and multidrug-resistant (MDR) isolates (Leite et al. 2025). The MAR index was calculated for each isolate as the ratio between the number of antimicrobial categories to which the isolate was resistant and the total number of antimicrobial categories evaluated. Oxacillin and cefoxitin were considered representatives of a single β-lactam category and were counted only once. MAR indices were summarized using the mean, median, minimum, maximum, and the proportion of isolates with values greater than 0.20 (Muwonge et al. 2025).

Resistance gene detection and biofilm production categories were also summarized as absolute and relative frequencies. Relationships among wild mammalian hosts, bacterial species, and anatomical sampling sites were explored graphically using count-based heat maps and bar plots. All statistical analyses and figures were produced using R software, version 4.3.1 (R Foundation for Statistical Computing, Vienna, Austria). No formal hypothesis testing was performed, and all findings were interpreted descriptively and exploratorily.

##  Results

###  Bacterial isolation and species identification

Confirmed bacterial isolates were recovered from 63 of the 84 wild mammals sampled. Because each animal contributed one oropharyngeal and one rectal swab, a total of 168 biological samples were processed. Only purified isolates confirmed as belonging to *Staphylococcus* spp. or *Mammaliicoccus sciuri* were retained in the final microbiological dataset. Confirmed isolates were recovered from 14 of the 15 host species sampled, whereas no target isolate was recovered from *Cavia aperea* (Table 3).

**Table 3:** Distribution of sampled wild mammals, animals yielding confirmed bacterial isolates, and isolates retained for microbiological analyses

Species	Sampled animals	Animals with positive bacterial cultures	Isolated bacteria
Cerdocyon thous	3/84 (3.6%)	3/3 (100%)	3/63 (4.8%)
Leopardus tigrinus	1/84 (1.2%)	1/1 (100%)	1/63 (1.6%)
Puma yagouaroundi	4/84 (4.8%)	4/4 (100%)	4/63 (6.3%)
Lontra longicaudis	1/84 (1.2%)	1/1 (100%)	1/63 (1.6%)
Procyon lotor	1/84 (1.2%)	1/1 (100%)	1/63 (1.6%)
Nasua nasua	3/84 (3.6%)	2/3 (66.7%)	2/63 (3.2%)
Euphractus sexcinctus	3/84 (3.6%)	3/3 (100%)	3/63 (4.8%)
Didelphis albiventris	27/84 (32.1%)	11/27 (40.7%)	11/63 (17.5%)
Bradypus variegatus	19/84 (22.6%)	19/19 (100%)	19/63 (30.2%)
Tamandua tetradactyla	6/84 (7.1%)	4/6 (66.7%)	4/63 (6.3%)
Sapajus libidinosus	2/84 (2.4%)	2/2 (100%)	2/63 (3.2%)
Callithrix jacchus	8/84 (9.5%)	8/8 (100%)	8/63 (12.7%)
Kerodon rupestris	1/84 (1.2%)	1/1 (100%)	1/63 (1.6%)
Coendou speratus	4/84 (4.8%)	3/4 (75.0%)	3/63 (4.8%)
Cavia aperea	1/84 (1.2%)	0/1 (0%)	0/63 (0%)

Each animal contributed two biological samples, one oropharyngeal swab and one rectal swab, totaling 168 swabs processed. Table 3 summarizes the host-level distribution of animals and the final set of confirmed Staphylococcus spp. and Mammaliicoccus sciuri isolates retained for microbiological analyses, rather than the total number of swabs cultured. Samples that did not yield recoverable target colonies, could not be purified, or were not confirmed within the target bacterial group were not retained in the final isolate dataset

**Fig. 1:**
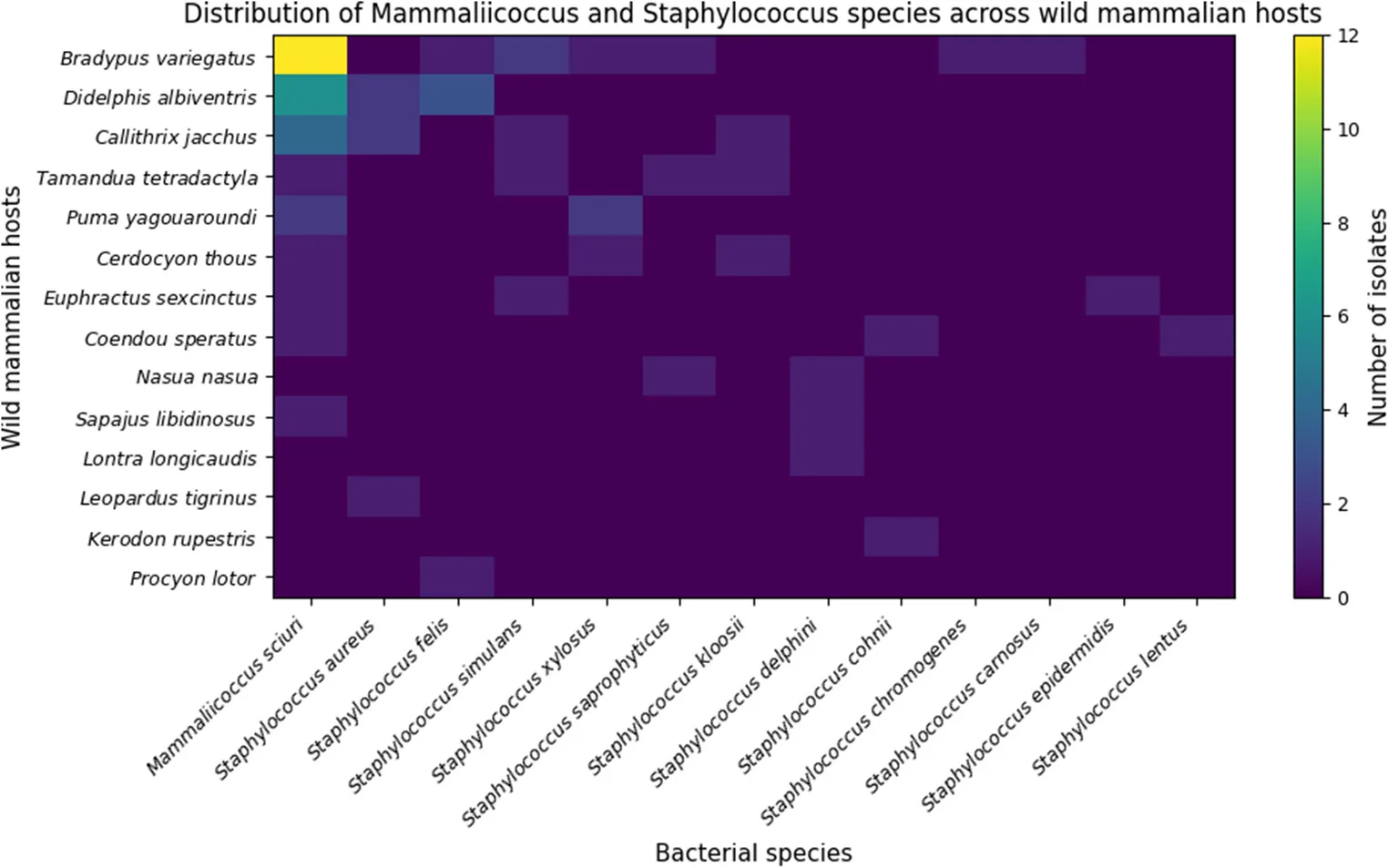
Distribution of *Mammaliicoccus* and *Staphylococcus* species isolated from wild mammals in northeastern Brazil Legend: Distribution of confirmed Mammaliicoccus and Staphylococcus isolates according to wild mammalian host species. The heat map represents the absolute number of isolates recovered for each host–bacterial species combination, with color intensity indicating isolate count. Host species and bacterial taxa were arranged according to their overall frequency of isolates to facilitate visualization of the main distribution patterns

Overall, 63 isolates were identified as belonging to the genera *Staphylococcus* and *Mammaliicoccus*. Of these, 29/63 (46.0%) were identified as *Mammaliicoccus sciuri*, while *Staphylococcus aureus*, *Staphylococcus felis*, and *Staphylococcus simulans* were each represented by 5/63 (7.9%) isolates. The distribution of bacterial species according to wild mammalian host species is shown in Fig. 1.

**Fig. 2:**
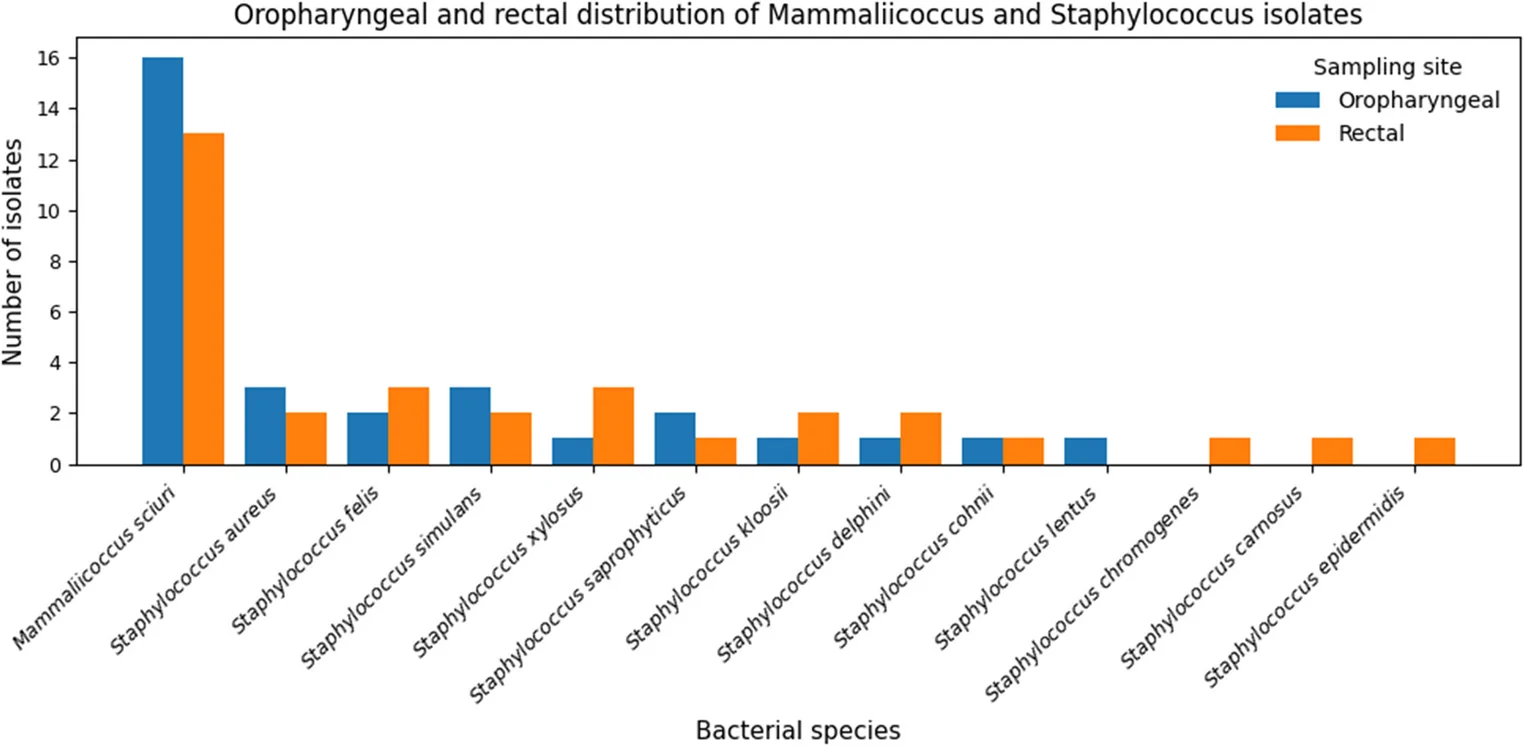
Distribution of anatomical sampling sites of *Mammaliicoccus and Staphylococcus species isolated from wild mammals*

Regarding anatomical origin, 31/63 isolates were recovered from oropharyngeal swabs and 32/63 from rectal swabs (Fig. 2; Table 4). Figure 2 shows the distribution of confirmed bacterial isolates according to anatomical sampling site, whereas Table 3 summarizes the host-level sampling structure and the number of confirmed isolates recovered from each wild mammal species.

**Table 4:** Distribution of antimicrobial-resistant isolates according to anatomical sampling site

Antimicrobial	Oropharyngeal, *n*/*N* (%)	Rectal, *n*/*N* (%)
Oxacillin	21/31 (67.7)	22/32 (68.8)
Cefoxitin	9/31 (29.0)	9/32 (28.1)
Norfloxacin	2/31 (6.5)	5/32 (15.6)
Gentamicin	3/31 (9.7)	4/32 (12.5)
Tetracycline	9/31 (29.0)	7/32 (21.9)
Clindamycin	7/31 (22.6)	7/32 (21.9)
Erythromycin	7/31 (22.6)	10/32 (31.3)
MDR	7/31 (22.6)	6/32 (18.8)

n/N number of resistant isolates/total number of isolates tested; MDR = multidrug resistance. MDR was defined as resistance to at least one antimicrobial agent in three or more antimicrobial categories, with oxacillin and cefoxitin considered within a single β-lactam category

###  Phenotypic antimicrobial resistance profiles

Overall, 50/63 (79.4%) isolates were resistant to at least one antimicrobial category, and 13/63 (20.6%) met the definition of multidrug resistance. For MDR classification, oxacillin and cefoxitin were considered representatives of the same β-lactam category and were therefore counted only once. Resistance to the β-lactam category was defined as resistance to oxacillin and/or cefoxitin.

Resistance to β-lactams was the most frequent phenotype and was observed in 45/63 (71.4%) isolates. When antimicrobial agents were considered individually, resistance was most frequently observed to oxacillin, in 43/63 (68.3%) isolates, followed by cefoxitin, in 18/63 (28.6%); erythromycin, in 17/63 (27.0%); tetracycline, in 16/63 (25.4%); clindamycin, in 14/63 (22.2%); norfloxacin, in 7/63 (11.1%); and gentamicin, in 7/63 (11.1%).

Resistance to at least one antimicrobial category was detected among isolates recovered from 13 of the 14 host species that yielded confirmed bacterial isolates. Among host species represented by at least three isolates, resistant isolates were recovered from *Bradypus variegatus* (17 isolates), *Didelphis albiventris* (9), *Callithrix jacchus* (4), *Puma yagouaroundi* (4), *Euphractus sexcinctus* (3), *Tamandua tetradactyla* (3), *Cerdocyon thous* (2), and *Coendou speratus* (2).

Multidrug-resistant isolates were recovered from *Bradypus variegatus* (3 isolates), *Callithrix jacchus* (2), *Didelphis albiventris* (2), *Euphractus sexcinctus* (2), and one isolate each from *Cerdocyon thous*, *Nasua nasua*, *Lontra longicaudis*, and *Leopardus tigrinus*. These findings are presented descriptively and should not be interpreted as prevalence estimates at the host-species level. The distribution of resistance to each antimicrobial agent according to bacterial species is presented in Table 5.

**Table 5:** Antimicrobial resistance profile of *Mammaliicoccus* and *Staphylococcus* isolates

Bacterial species	*n*	OXA	CFO	NOR	GEN	TET	CLI	ERI
*Mammaliicoccus sciur*	29	22/29 (75.9%)	11/29 (37.9%)	2/29 (6.9%)	4/29 (13.8%)	6/29 (20.7%)	7/29 (24.1%)	7/29 (24.1%)
*Staphylococcus aureus*	5	4/5 (80.0%)	2/5 (40.0%)	1/5 (20.0%)	0/5 (0.0%)	1/5 (20.0%)	0/5 (0.0%)	2/5 (40.0%)
*Staphylococcus carnosus*	1	1/1 (100.0%)	1/1 (100.0%)	1/1 (100.0%)	1/1 (100.0%)	1/1 (100.0%)	1/1 (100.0%)	1/1 (100.0%)
*Staphylococcus chromogenes*	1	1/1 (100.0%)	0/1 (0.0%)	0/1 (0.0%)	0/1 (0.0%)	0/1 (0.0%)	0/1 (0.0%)	0/1 (0.0%)
*Staphylococcus cohnii*	2	(50.0%)	0/2 (0.0%)	0/2 (0.0%)	0/2 (0.0%)	0/2 (0.0%)	0/2 (0.0%)	0/2 (0.0%)
*Staphylococcus delphini*	3	2/3 (66.7%)	1/3 (33.3%)	1/3 (33.3%)	1/3 (33.3%)	1/3 (33.3%)	1/3 (33.3%)	1/3 (33.3%)
*Staphylococcus epidermidis*	1	0/1 (0.0%)	0/1 (0.0%)	0/1 (0.0%)	0/1 (0.0%)	0/1 (0.0%)	1/1 (100.0%)	1/1 (100.0%)
*Staphylococcus felis*	5	2/5 (40.0%)	0/5 (0.0%)	0/5 (0.0%)	0/5 (0.0%)	1/5 (20.0%)	1/5 (20.0%)	1/5 (20.0%)
*Staphylococcus kloosii*	3	1/3 (33.3%)	0/3 (0.0%)	0/3 (0.0%)	0/3 (0.0%)	0/3 (0.0%)	0/3 (0.0%)	0/3 (0.0%)
*Staphylococcus lentus*	1	1/1 (100.0%)	0/1 (0.0%)	0/1 (0.0%)	0/1 (0.0%)	0/1 (0.0%)	0/1 (0.0%)	0/1 (0.0%)
*Staphylococcus saprophyticus*	3	2/3 (66.7%)	1/3 (33.3%)	1/3 (33.3%)	0/3 (0.0%)	2/3 (66.7%)	2/3 (66.7%)	2/3 (66.7%)
*Staphylococcus simulans*	5	3/5 (60.0%)	1/5 (20.0%)	0/5 (0.0%)	1/5 (20.0%)	2/5 (40.0%)	0/5 (0.0%)	1/5 (20.0%)
*Staphylococcus xylosus*	4	3/4(75.0%)	1/4 (25.0%)	1/4 (25.0%)	0/4 (0.0%)	2/4 (50.0%)	1/4 (25.0%)	1/4 (25.0%)

*OXA* oxacillin, *CFO* cefoxitin, *NOR* norfloxacin, *GEN* gentamicin, *TET* tetracycline, *CLI* clindamycin, *ERI* erythromycin. For *MDR* and *MAR* index calculations, *OXA* and CFO were considered representatives of the β-lactam category and were counted only once. Results for bacterial species represented by one or two isolates should be interpreted descriptively, without statistical inference or generalization at the species level

*Mammaliicoccus sciuri* was the most frequently identified species, accounting for 29/63 (46.0%) isolates. Resistance to at least one antimicrobial category was detected in 25/29 (86.2%) *M. sciuri* isolates, and 5/29 (17.2%) were classified as MDR. 

Among *Staphylococcus* species, resistance to at least one antimicrobial category was detected in all isolates of *S. aureus* (5/5), *S. saprophyticus* (3/3), and *S. xylosus* (4/4), as well as in 2/3 *S. delphini* isolates and 3/5 *S. simulans* isolates. MDR was identified in 1/5 *S. aureus*, 2/3 *S. saprophyticus*, 1/4 *S. xylosus*, 1/3 *S. delphini*, and 1/5 *S. simulans* isolates. Resistance to at least one antimicrobial category was also observed in the single isolates of *S. carnosus*, *S. chromogenes*, *S. epidermidis*, and *S. lentus*. Because several bacterial species were represented by only one or a few isolates, these findings should be interpreted descriptively and should not be generalized at the species level.

Overall, MAR index values, calculated by antimicrobial category and considering oxacillin and cefoxitin as a single β-lactam category, ranged from 0.00 to 1.00. The mean MAR index was 0.28, the median was 0.17, and 25/63 (39.7%) isolates had MAR index values greater than 0.20.

###  Detection of antimicrobial resistance genes

The *mecA* gene was detected in 4/63 (6.3%) isolates, including two *Mammaliicoccus sciuri* isolates recovered from one *Bradypus variegatus* and one *Didelphis albiventris* individual, one *Staphylococcus delphini* isolate recovered from a *Sapajus libidinosus* individual, and one *Staphylococcus aureus* isolate recovered from a *Didelphis albiventris* individual. All *mecA*-positive isolates were phenotypically resistant to cefoxitin. Two of these isolates exhibited multidrug resistance and had MAR index values greater than 0.20.

The *mecC* gene was not detected in any isolate. The efflux pump gene *norA* was detected in 2/63 (3.2%) *M. sciuri* isolates recovered from *Sapajus libidinosus* and *Didelphis albiventris*. Both *norA*-positive isolates were phenotypically susceptible to norfloxacin; therefore, the presence of *norA* was not associated with phenotypic norfloxacin resistance in the present dataset.

The *msrA* gene was detected in a single *S. delphini* isolate recovered from *Sapajus libidinosus*. This isolate exhibited an intermediate susceptibility phenotype to erythromycin. In contrast, *tet(38)* and *tetM* were not detected, despite the occurrence of phenotypic tetracycline resistance among the isolates.

###  Biofilm formation capacity

Overall, 33/63 (52.4%) isolates exhibited biofilm-forming capacity, whereas 30/63 (47.6%) were classified as non-producers. Among all isolates, 23/63 (36.5%) were classified as weak biofilm producers, 8/63 (12.7%) as moderate producers, and 2/63 (3.2%) as strong producers.

*Mammaliicoccus sciuri* accounted for most biofilm-forming isolates, with 23/29 (79.3%) isolates classified as producers. These included 17/29 (58.6%) weak producers, 5/29 (17.2%) moderate producers, and 1/29 (3.4%) strong producer.

Biofilm formation was also observed in 2/3 (66.7%) *S. saprophyticus* isolates, 1/2 (50.0%) *S. cohnii* isolates, 2/5 (40.0%) isolates each of *S. aureus* and *S. felis*, 1/4 (25.0%) *S. xylosus* isolates, and 1/5 (20.0%) *S. simulans* isolates. Among the two biofilm-producing *S. aureus* isolates, one was classified as a weak producer and the other as a moderate producer.

The single *S. chromogenes* isolate was classified as a weak biofilm producer. In contrast, all isolates of *S. delphini*, *S. kloosii*, *S. lentus*, *S. carnosus*, and *S. epidermidis* were classified as non-producers.

Descriptively, no clear pattern was observed between biofilm formation category and multidrug resistance. MDR was detected in 8/30 (26.7%) biofilm-negative isolates and 5/23 (21.7%) weak biofilm producers, whereas none of the moderate or strong biofilm producers exhibited MDR. Consistent with this distribution, mean MAR index values were highest among biofilm-negative isolates (0.32), followed by weak (0.27), moderate (0.21), and strong biofilm producers (0.08).

##  Discussion

This study combined phenotypic and genotypic antimicrobial resistance analyses, multidrug resistance classification, MAR indices, phenotype–genotype comparisons, and biofilm assessment in *Staphylococcus* spp. and *Mammaliicoccus sciuri* recovered from wild mammals undergoing rehabilitation.

The main findings were the frequent recovery of *M. sciuri*, the predominance of β-lactam resistance, the occurrence of multidrug-resistant isolates, the detection of selected resistance genes, and biofilm formation in approximately half of the isolates. Together, these findings provide baseline microbiological evidence supporting the inclusion of wildlife rehabilitation facilities in antimicrobial resistance surveillance.

The results should be interpreted considering the study design. The uneven and limited number of animals per host species restricts host-specific comparisons, while the opportunistic and cross-sectional sampling prevents conclusions regarding causality, transmission routes, or whether resistant bacteria were acquired before or during rehabilitation.

Information concerning animal origin, previous captivity, antimicrobial exposure, housing, diet, and length of stay was not uniformly available. Furthermore, the study was restricted to a single rehabilitation center and did not include environmental, personnel, longitudinal, or genomic sampling. Therefore, the findings represent descriptive microbiological evidence rather than confirmation of ecological patterns, reservoir status, or transmission dynamics.

*Mammaliicoccus sciuri* was the most frequently recovered species, representing 46.0% of the isolates. Members of the *M. sciuri* group are recognized as colonizers and opportunistic bacteria and may harbor antimicrobial resistance determinants relevant to other staphylococcal species (Becker et al. 2014; De Carvalho et al. 2024).

The bacterial diversity observed also included *S. aureus*, *S. felis*, *S. delphini*, *S. simulans*, and several coagulase-negative staphylococci. Some of these species have recognized opportunistic or zoonotic relevance. *Staphylococcus aureus* has been associated with host switching and transmission between humans and animals, while *S. felis* and *S. delphini* have occasionally been implicated in human infections following animal exposure (Heaton et al. 2020; Magleby et al. 2019; Sips et al. 2023).

Coagulase-negative staphylococci may also carry mobile antimicrobial resistance determinants and contribute to their persistence within microbial communities (Becker et al. 2014). Future genomic investigations should evaluate strain diversity, virulence markers, and mobile genetic elements among these wildlife-associated isolates.

The frequent recovery of *M. sciuri* also highlights the potential influence of the microbiological procedures used. Mannitol Salt Agar and the saline enrichment conditions applied in this study favor the recovery of halotolerant bacteria and may have affected the observed species distribution (Parker et al. 2024).

Consequently, the relative frequency of *M. sciuri* should not be interpreted as evidence of host adaptation or ecological predominance. Future studies combining selective and non-selective culture media with culture-independent approaches could provide a broader characterization of the bacterial communities associated with wild mammals undergoing rehabilitation (Parker et al. 2024; Vezeau and Kahn 2024).

The predominance of β-lactam resistance and the occurrence of multidrug-resistant isolates were the main phenotypic resistance findings. European studies provide relevant, although not directly comparable, contexts. Cagnoli et al. (2024) investigated nasal staphylococci exclusively in fallow deer (*Dama dama*) from Italy, whereas González-Azcona et al. (2025) evaluated the nasal bacteriome of European wild rabbits (*Oryctolagus cuniculus*) from Spain and Portugal. Mateus-Vargas et al. (2022) examined Staphylococcaceae with reduced cefoxitin susceptibility in four species of wild ungulates in Germany.

Differences in host species, geographical regions, anatomical sampling sites, culture methods, antimicrobial panels, and interpretative criteria limit direct numerical comparisons. Nevertheless, these studies demonstrate that resistance among wildlife-associated staphylococci may vary according to ecological and methodological contexts (Mateus-Vargas et al. 2022; Cagnoli et al. 2024; González-Azcona et al. 2025). Harmonized antimicrobial panels and interpretative criteria would improve comparisons among wildlife studies conducted in different regions.

Beyond methodological differences, the ecological characteristics of the sampled hosts generate specific hypotheses for future investigation. *Callithrix jacchus* and *Cerdocyon thous* may use urban or human-modified environments, whereas *Lontra longicaudis* is associated with aquatic habitats that may receive anthropogenic inputs (Albuquerque and Oliveira 2020; Rheingantz et al. 2017; Swift et al. 2019).

The recovery of resistant isolates from *Bradypus variegatus* and *Euphractus sexcinctus* also raises questions regarding the influence of habitat use, diet, contact with soil or water, and proximity to urban environments (Dalponte and Tavares-Filho 2004; Urbani and Bosque 2007). Future studies should test whether resistance profiles are associated with landscape anthropization, host behavior, habitat type, reason for admission, previous antimicrobial exposure, or duration of rehabilitation.

Original studies from South America support the investigation of these hypotheses. Antimicrobial resistance genes have been associated with anthropized landscapes in guignas (*Leopardus guigna*) and Andean foxes (*Lycalopex culpaeus*) from Chile (Sacristán et al. 2020; Cevidanes et al. 2020). Differences in resistance gene occurrence have also been identified between Brazilian seabird species with contrasting ecological behaviors (Ewbank et al. 2021).

In addition, antimicrobial-resistant bacteria have been detected in animals and environmental surfaces within a wildlife rehabilitation center in Chile, although direct transmission between these compartments was not demonstrated (Baros Jorquera et al. 2021). These findings indicate that ecological, clinical, and environmental variables should be evaluated jointly to determine which factors influence antimicrobial resistance in rehabilitating Neotropical wildlife.

The detection of *mecA* in *M. sciuri*, *S. delphini*, and *S. aureus* demonstrates the occurrence of a clinically relevant methicillin-resistance determinant in different bacterial species within the analyzed collection. The occurrence of *mecA* in South American wildlife has previously been reported in guignas and Andean foxes from Chile, particularly in animals exposed to anthropized environments (Sacristán et al. 2020; Cevidanes et al. 2020). All *mecA*-positive isolates in the present study were resistant to cefoxitin, indicating agreement between the molecular and phenotypic findings for these isolates.

In contrast, *mecC* was not detected and therefore did not explain the remaining cefoxitin-resistant phenotypes. Fourteen of the 18 cefoxitin-resistant isolates were negative for both *mecA* and *mecC*. This phenotype–genotype discordance may involve resistance determinants not investigated in the PCR panel, alterations in penicillin-binding proteins, other *mec* homologues, or limitations associated with applying common interpretative criteria to different staphylococcal and mammaliicoccal species (Becker et al. 2014; Brdová et al. 2024). Broader molecular panels and whole-genome sequencing are needed to clarify the mechanisms underlying cefoxitin resistance in these isolates.

Tetracycline resistance was also detected despite the absence of *tet(38)* and *tetM*. Other determinants, particularly *tet(K)* and *tet(L)*, may contribute to efflux-mediated tetracycline resistance in staphylococci and should be investigated in future studies (Matuschek et al. 2014; Brdová et al. 2024). Similarly, the two *norA*-positive isolates were susceptible to norfloxacin, while the single *msrA*-positive isolate presented an intermediate erythromycin phenotype. The presence of a resistance gene does not necessarily result in phenotypic resistance because gene expression, regulatory mechanisms, mutations, and antimicrobial-specific interpretative criteria may influence the observed phenotype (Becker et al. 2014; Feßler et al. 2018). Future analyses combining gene detection, sequencing, and expression assays could clarify these discrepancies.

Biofilm formation was observed in approximately half of the isolates, predominantly among weak and moderate producers. No consistent association was observed between biofilm production and multidrug resistance, since MDR isolates occurred only among non-producers and weak producers. Previous studies have shown that the relationship between antimicrobial resistance and biofilm formation is variable and may depend on bacterial species, strain, growth conditions, and the methods used for biofilm classification (Feßler et al. 2018; Gajdács et al. 2021). Thus, within the present isolate collection, biofilm formation and multidrug resistance appeared to represent distinct bacterial characteristics rather than directly associated traits.

The interpretation of the biofilm findings should also consider the reference strain used. *Escherichia coli* ATCC 35,218 has been described as a weak biofilm producer and was used as a procedural control for bacterial adherence and crystal-violet staining rather than as a reference for strong biofilm production (Gajdács et al. 2021). Therefore, the assay did not include a well-characterized strong biofilm-producing strain capable of validating the full analytical range of the classification system. Future studies should include a strong biofilm-producing reference strain, preferably belonging to the genus *Staphylococcus*, and investigate genes associated with adhesion and biofilm development.

Comparisons with previous wildlife studies should also account for methodological and biological differences. Baros Jorquera et al. (2021) investigated mainly Gram-negative bacteria with reduced susceptibility to cephalosporins in animals and environmental surfaces from a Chilean rehabilitation center. In contrast, the present study focused on *Staphylococcus* spp. and *M. sciuri* obtained from oropharyngeal and rectal samples and combined disk diffusion, targeted PCR, and phenotypic biofilm assessment without environmental sampling. European investigations involving ungulates and rabbits also differed in host populations, anatomical sites, culture media, antimicrobial panels, and molecular approaches (Mateus-Vargas et al. 2022; Cagnoli et al. 2024; González-Azcona et al. 2025). These distinctions define the contribution of the present study as a characterization of resistant and biofilm-forming Gram-positive bacteria in rehabilitating Neotropical mammals.

The practical implications of the findings apply to the confirmed isolates recovered from 63 animals representing 14 host species and should not be generalized to all animals housed at the center or to unsampled wildlife populations. Nevertheless, the detection of resistant, multidrug-resistant, gene-positive, and biofilm-forming isolates supports the inclusion of wildlife rehabilitation facilities in antimicrobial resistance surveillance (Baros Jorquera et al. 2021).

These centers bring together animals from different origins, veterinary personnel, shared equipment, enclosures, and clinical procedures, creating potential opportunities for microbial exchange that should be investigated through integrated sampling (Sánchez-Ortiz et al. 2024).

Preventive measures should include prudent antimicrobial use, microbiological testing when clinically indicated, standardized cleaning and disinfection, hand hygiene, dedicated or appropriately disinfected equipment, and procedures that reduce cross-contamination during animal handling. Antimicrobial stewardship protocols adapted to the clinical and logistical realities of wildlife rehabilitation facilities may help guide therapeutic decisions and biosafety practices (Parker et al. 2024; Weese et al. 2024).

Future surveillance should incorporate standardized sampling at admission, during prolonged hospitalization, and before release. Individual records should systematically include the animal’s origin, reason for admission, prior captivity, previous antimicrobial exposure, enclosure history, clinical procedures, and length of stay (Baros Jorquera et al. 2021; Parker et al. 2024). Whenever operationally feasible, animal sampling should be combined with environmental and personnel sampling, isolate preservation, and genomic analyses. Multicenter and longitudinal studies using harmonized antimicrobial panels would help determine whether resistant bacteria are introduced at admission, acquired during rehabilitation, or exchanged among animals and the care environment (Baros Jorquera et al. 2021; Parker et al. 2024; Vezeau and Kahn 2024).

Overall, this study provides baseline evidence of antimicrobial resistance, selected resistance genes, and biofilm formation among *Staphylococcus* spp. and *M. sciuri* recovered from wild mammals undergoing rehabilitation. Its main contribution is not the demonstration of transmission or reservoir status, but the identification of microbiological findings that justify structured surveillance, broader molecular investigations, and biosafety-oriented research at the human–animal–environment interface.

## Conclusion

These results provide microbiological baseline evidence supporting the inclusion of wildlife rehabilitation facilities in antimicrobial resistance surveillance efforts and reinforce the importance of biosafety-oriented practices, prudent antimicrobial use, and infection-control measures in these settings. Although the present data do not establish transmission pathways, reservoir status, or formal bioindicator roles, they highlight wildlife care facilities as relevant operational contexts for future integrated investigations at the human–animal–environment interface.

## Supplementary material

Below is the link to the electronic supplementary material.

Supplementary Material 1

## Data Availability

The datasets generated and/or analysed during the current study are available from the corresponding author on reasonable request.
